# Evaluating Angiogenic Biomarkers in Alzheimer's Disease: A Systematic Review and Meta‐Analysis

**DOI:** 10.1002/alz70856_107021

**Published:** 2026-01-08

**Authors:** Arunima Kapoor, Jillian L. Joyce, Amy Nguyen, Aimée Gaubert, S. Duke Han, Christopher C. W. Hughes, Daniel A. Nation

**Affiliations:** ^1^ University of California, Irvine, Irvine, CA, USA; ^2^ University of Southern California, Los Angeles, CA, USA; ^3^ University of Southern California, Leonard Davis School of Gerontology, Los Angeles, CA, USA; ^4^ Leonard Davis School of Gerontology, University of Southern California, Los Angeles, CA, USA; ^5^ University Of California, Irvine, Irvine, CA, USA; ^6^ Keck School of Medicine, University of Southern California, Los Angeles, CA, USA

## Abstract

**Background:**

Cerebrovascular dysfunction can contribute to the pathophysiology of Alzheimer's disease (AD) and trigger angiogenesis. Studies examining angiogenic biomarkers in AD have yielded conflicting results. No prior systematic review and meta‐analysis has qualitatively and quantitatively evaluated existing literature examining angiogenic biomarkers in AD. In this review, we aimed to identify markers of angiogenesis in biofluids and brain tissue that can differentiate between individuals with AD and healthy older adults, and inform the role of angiogenesis in AD.

**Method:**

Using Medline (1946 to August 04, 2021), the literature was systematically searched for articles according to PRISMA guidelines. Angiogenesis and AD terms were searched as keywords and mapped to MeSH headings. A total of 2203 records were screened; 209 studies were assessed for eligibility, and 16 studies (including 25 dependent effect sizes) were included. Hedges’ *g* was selected as the effect size of interest to determine the standardized difference in angiogenesis protein markers between AD and control groups in biofluids. Neuropathological studies were reviewed qualitatively.

**Result:**

A random‐effects model including all eligible studies revealed a small and non‐significant overall effect size (combined Hedges’ *g* = −0.14, *p* = 0.792), with significant heterogeneity (I^2^ = 99.35%, Q‐statistic *p* < .001). Fourteen of 25 effect sizes were derived from studies examining vascular endothelial growth factor (VEGF) markers and demonstrated significant heterogeneity. Eight neuropathological studies demonstrated elevated levels of VEGF in AD brains, while two showed reductions in VEGF expression. Four studies utilizing fibroblast growth factor (FGF) or platelet derived growth factor (PDGF) associated markers in biofluids differentiated AD from healthy controls. Four neuropathological studies showed reduced pericyte PDGFRβ in AD brains and two found a corresponding increase in the ligand PDGF‐BB levels.

**Conclusion:**

Despite being the most studied marker, VEGF levels were not significantly different in AD and normal aging. Few studies have examined FGF and PDGFRβ/BB; these markers warrant further investigation. Significant heterogeneity among studies suggests that improvements in methods, including reliable assays, are needed. Identifying novel angiogenic biomarkers could elucidate the role of vascular mechanisms in AD and reveal potential therapeutic targets.

